# Supplementary material for: GSTA4 mediates reduction of cisplatin ototoxicity in female mice
Source: Nat Commun. 2019 Sep 12;10:4150. doi: 10.1038/s41467-019-12073-0 (PMC6742643; doi:10.1038/s41467-019-12073-0)
Supplement: Supplementary file 1 — Supplementary Information [file 41467_2019_12073_MOESM1_ESM.pdf]

Supplementary Information

GSTA4 Mediates Reduction of Cisplatin Ototoxicity in Female Mice

Hyo-Jin Park[1,9], Mi-Jung Kim[1,9], Christina Rothenberger[1,9], Ashok Kumar[2], Edith M. Sampson[3], Dalian Ding[4], Chul Han[1], Karessa White[1], Kevin Boyd[1], Manohar Senthilvelan[4], Yong-Hwan Kim[5], Maria S. Ticsa[1], Aaron S. Gomez[1], Isabela Caicedo[1], Upal Bose[1], Paul J. Linser[6], Takuya Miyakawa[7], Masaru Tanokura[7], Thomas C. Foster[2], Richard Salvi[4,8], and Shinichi Someya[1,*]

[1]Department of Aging and Geriatric Research, University of Florida, Gainesville, FL, 32610, USA.
[2]Department of Neuroscience, University of Florida, Gainesville, FL, 32611, USA.
[3]Monoclonal Antibody Core, Interdisciplinary Center for Biotechnology Research, University of Florida, Gainesville, FL, 32610, USA
[4]Center for Hearing and Deafness, State University of New York at Buffalo, NY, 14214, USA.
[5]Department of Neurobiology, Barrow Neurological Institute, Phoenix, AZ, 85013, USA
[6]Whitney Laboratory, University of Florida, St Augustine, FL, 32080, USA.
[7]Department of Applied Biological Chemistry, University of Tokyo, Yayoi, Tokyo, 113-8657, Japan.
[9]These authors contributed equally to this work.

*Correspondence and request for materials should be addressed to S. S. (someya@ufl.edu)

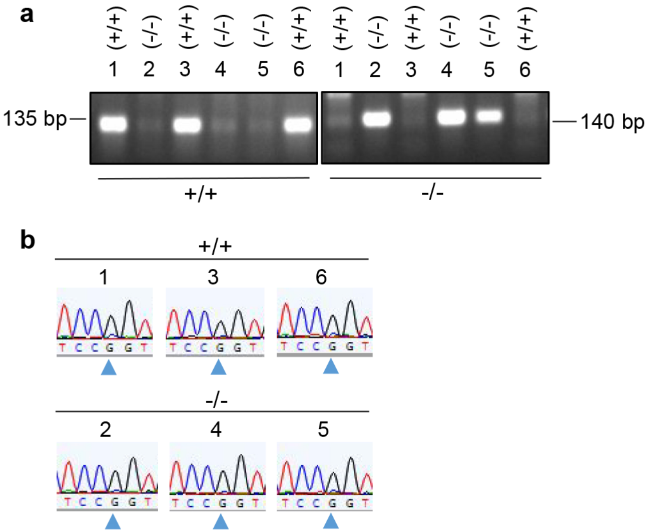

Supplementary Fig. 1. Genotyping of *Gsta4*$^{+/+}$, *Gsta4*$^{+/-}$, and *Gsta4*$^{-/-}$ mice. **a** PCR products were separated on a 1.5% agarose gel. The expected band sizes for the wild-type and mutant alleles were 135 and 140 bps, respectively. The full-length gel is presented in the Source Data file. **b** The *Cdh23* gene in three *Gsta4*$^{+/+}$ and three *Gsta4*$^{-/-}$ mice was sequenced. All the mice examined had the same wild-type *Cdh23*$^{753G/753G}$ genotype.

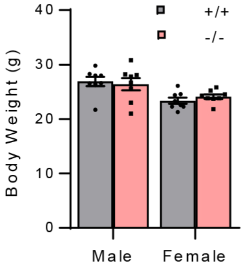

Supplementary Fig. 2. Assessment of body weight. The body weight of male and female *Gsta4*$^{+/+}$ and *Gsta4*$^{-/-}$ mice was measured at 3 months of age. *n*=8, error bars represent ± s.e.m. Source data are provided as a Source Data file.

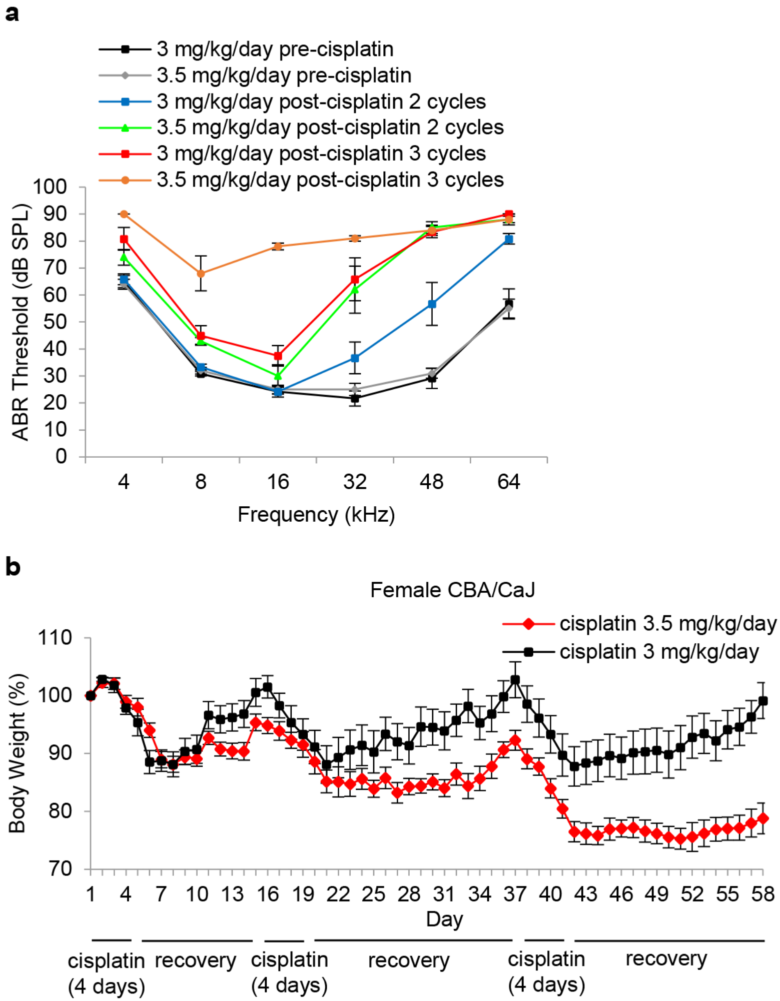

Supplementary Fig. 3. Assessment of ABR hearing thresholds. **a** ABR hearing thresholds were measured at 4, 8, 16, 32, 48 and 64 kHz prior to, after 2 cycles or 3 cycles of cisplatin treatment at 3.0 or 3.5 mg/kg/day of cisplatin. The quantification shows mean of at least five independent experiments ($n = 5$), error bars represent ± s.e.m. **b** The body weight of female CBA/CaJ mice was measured during cisplatin treatment. The quantification shows mean of at least five independent experiments ($n = 5$), error bars represent ± s.e.m. Source data are provided as a Source Data file.

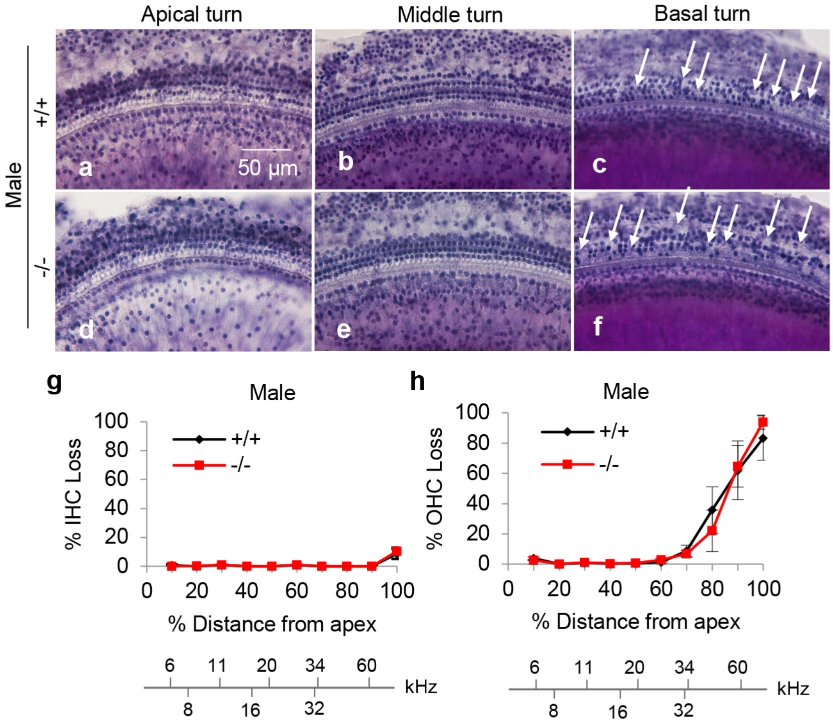

Supplementary Fig. 4. Cochleograms. **a-f**: Hair cell regions in the apical, middle and basal regions of cochlear basilar membranes from male *Gsta4*$^{+/+}$ and *Gsta4*$^{-/-}$ mice after cisplatin treatment. Arrows indicate missing hair cells. Scale bar = 50 µm. **g-h**: Cochleograms were recorded and averaged in the cochlear tissues of male *Gsta4*$^{+/+}$ and *Gsta4*$^{-/-}$ mice. Graphs show percent loss of IHCs (g) and OHCs (h) as function of percent distance from the apex. The quantification shows mean of at least five independent experiments ($n = 4$), error bars represent ± s.e.m. Lower x-axes show the frequency-place map for the mouse cochlea. Source data are provided as a Source Data file.

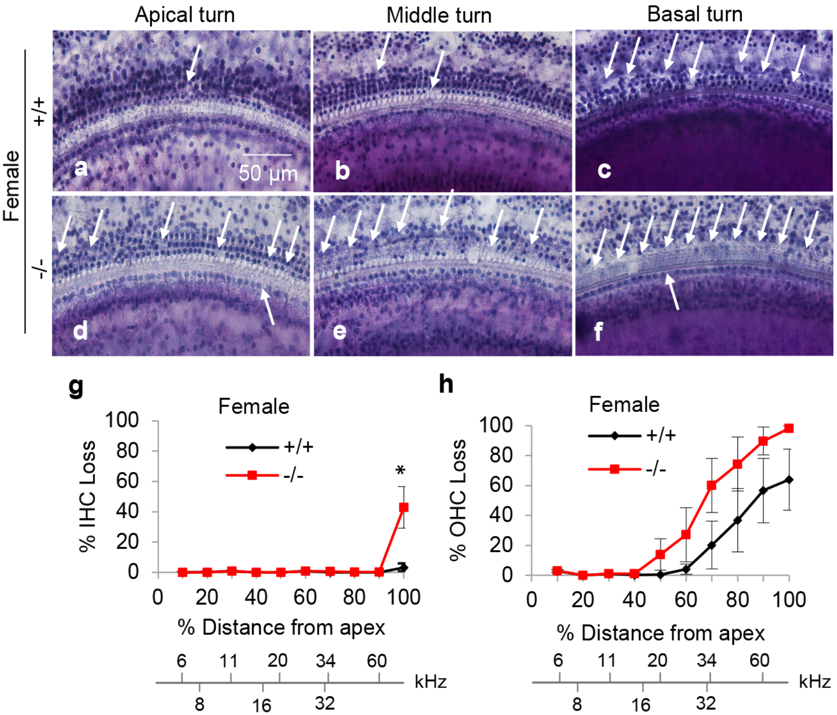

Supplementary Fig. 5. Cochleograms. **a-f**: Hair cell regions in the apical, middle and basal regions of cochlear basilar membranes from female *Gsta4*$^{+/+}$ and *Gsta4*$^{-/-}$ mice after cisplatin treatment. Arrows indicate missing hair cells. Scale bar = 50 µm. **g-h**: Cochleograms were recorded and averaged in the cochlear tissues of female *Gsta4*$^{+/+}$ and *Gsta4*$^{-/-}$ mice. Graphs show percent loss of IHCs (g) and OHCs (h) as function of percent distance from the apex. The quantification shows mean of at least four independent experiments ($n = 4$), *$p < 0.05$ vs. +/+ (two-way ANOVA), error bars represent ± s.e.m. Lower x-axes show the frequency-place map for the mouse cochlea. Source data are provided as a Source Data file.

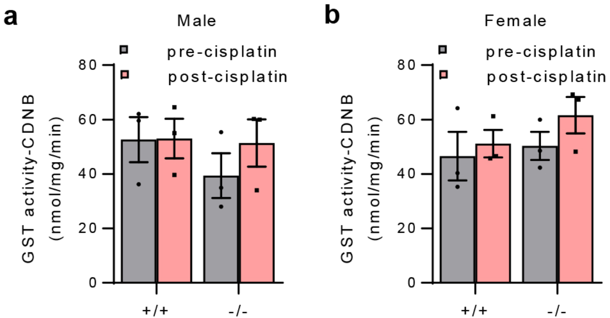

Supplementary Fig. 6. Assessment of GST activity. **a-b** GST activities toward CDNB were measured in the cytosol of inner ear tissues from males (**a**) and female (**b**) *Gsta4*$^{+/+}$ and *Gsta4*$^{-/-}$ mice prior to and after cisplatin treatment. n=3, error bars represent ± s.e.m. Source data are provided as a Source Data file.

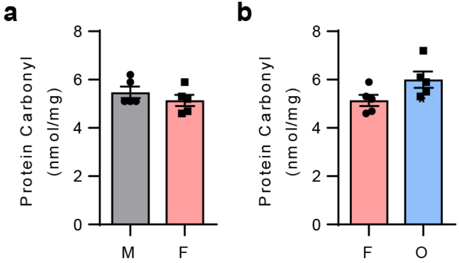

Supplementary Fig. 7. Assessment of oxidative protein damage. **a-b** Levels of protein carbonyl as an oxidative protein damage marker were measured in the inner ear tissues from male, female, and ovariectomized female *Gsta4*$^{-/-}$ mice. n=5, error bars represent ± s.e.m. Source data are provided as a Source Data file.

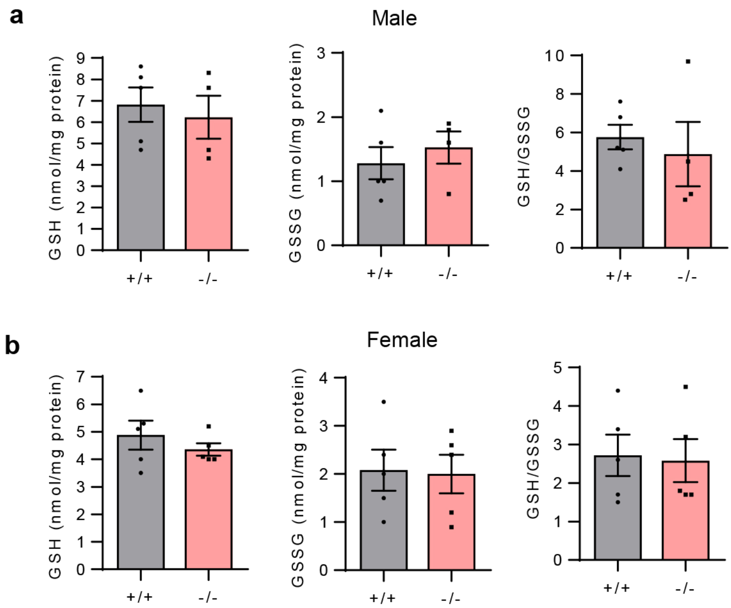

Supplementary Fig. 8. Assessment of glutathione redox status. **a-b:** Levels of GSH (left), GSSG (middle), and GSH/GSSG (right) were measured in the cytosol of inner ear tissues from males (**a**) and female (**b**) *Gsta4*$^{+/+}$ and *Gsta4*$^{-/-}$ mice after cisplatin treatment. The quantification shows mean of at least five independent experiments ($n = 4$), error bars represent $\pm$ s.e.m. Source data are provided as a Source Data file.
