# Supplementary material for: GSTA4 mediates reduction of cisplatin ototoxicity in female mice
Source: Nat Commun. 2019 Sep 12;10:4150. doi: 10.1038/s41467-019-12073-0 (PMC6742643; doi:10.1038/s41467-019-12073-0)
Supplement: Supplementary file 3 — Reporting Summary [file 41467_2019_12073_MOESM3_ESM.pdf]

nature research

Corresponding author(s): Shinichi Someya

Last updated by author(s): Jul 15, 2019

Reporting Summary

Nature Research wishes to improve the reproducibility of the work that we publish. This form provides structure for consistency and transparency in reporting. For further information on Nature Research policies, see Authors & Referees and the Editorial Policy Checklist.

Statistics

For all statistical analyses, confirm that the following items are present in the figure legend, table legend, main text, or Methods section.

n/a	Confirmed	
☐	☒	The exact sample size (n) for each experimental group/condition, given as a discrete number and unit of measurement
☐	☒	A statement on whether measurements were taken from distinct samples or whether the same sample was measured repeatedly
☐	☒	The statistical test(s) used AND whether they are one- or two-sided<br>*Only common tests should be described solely by name; describe more complex techniques in the Methods section.*
☒	☐	A description of all covariates tested
☒	☐	A description of any assumptions or corrections, such as tests of normality and adjustment for multiple comparisons
☒	☐	A full description of the statistical parameters including central tendency (e.g. means) or other basic estimates (e.g. regression coefficient) AND variation (e.g. standard deviation) or associated estimates of uncertainty (e.g. confidence intervals)
☒	☐	For null hypothesis testing, the test statistic (e.g. F, t, r) with confidence intervals, effect sizes, degrees of freedom and P value noted<br>*Give P values as exact values whenever suitable.*
☒	☐	For Bayesian analysis, information on the choice of priors and Markov chain Monte Carlo settings
☒	☐	For hierarchical and complex designs, identification of the appropriate level for tests and full reporting of outcomes
☒	☐	Estimates of effect sizes (e.g. Cohen's d, Pearson's r), indicating how they were calculated

Our web collection on statistics for biologists contains articles on many of the points above.

Software and code

Policy information about availability of computer code

Data collection	All data collection used standard procedures as described in methods.
Data analysis	All data analysis steps and programs were standard, published before and are described in methods.

For manuscripts utilizing custom algorithms or software that are central to the research but not yet described in published literature, software must be made available to editors/reviewers. We strongly encourage code deposition in a community repository (e.g. GitHub). See the Nature Research guidelines for submitting code & software for further information.

Data

Policy information about availability of data

All manuscripts must include a data availability statement. This statement should provide the following information, where applicable:

- Accession codes, unique identifiers, or web links for publicly available datasets
- A list of figures that have associated raw data
- A description of any restrictions on data availability

All data supporting the findings of this study are available within the article (and its Supplementary Information file and Source Data file) or from the corresponding author upon request.

Field-specific reporting

Please select the one below that is the best fit for your research. If you are not sure, read the appropriate sections before making your selection.

☒ Life sciences ☐ Behavioural & social sciences ☐ Ecological, evolutionary & environmental sciences

Life sciences study design

All studies must disclose on these points even when the disclosure is negative.

Sample size	Initial sample sizes were determined based on biological variance observed in previous ABR experiments and histochemical quantifications.
Data exclusions	Mice that failed to gain weight or if their BW did not rises to above 75% at the end of each 10-day recover period after cisplatin treatment were euthanized and excluded from the analyses. Final sample sizes are indicated in the figure legends.
Replication	Critical experiments with a statistically significant outcome were replicated successfully.
Randomization	For each experiment, mice were age- and sex-matched and randomly assigned to groups
Blinding	Blinding was not necessary for cisplatin treatment experiments as we were required to work with Animal Care Services Veterinarians on the cisplatin-treated mice: any cisplatin-treated mice that displayed inadequate hydration or any mouse whose body weight exceeded 15% difference from that of an aged-matched control mouse were put on veterinary surveillance.

Reporting for specific materials, systems and methods

We require information from authors about some types of materials, experimental systems and methods used in many studies. Here, indicate whether each material, system or method listed is relevant to your study. If you are not sure if a list item applies to your research, read the appropriate section before selecting a response.

Materials & experimental systems

n/a	Involved in the study
☐	☒ Antibodies
☒	☐ Eukaryotic cell lines
☒	☐ Palaeontology
☐	☒ Animals and other organisms
☒	☐ Human research participants
☒	☐ Clinical data

Methods

n/a	Involved in the study
☒	☐ ChIP-seq
☒	☐ Flow cytometry
☒	☐ MRI-based neuroimaging

Antibodies

Antibodies used	All antibodies used were described in methods.
Validation	All antibodies were standard from commercial vendors. Tissue stained without primary antibody and/or Gsta4 KO tissues served to rule out the possibility of non-specific binding of secondary antibody to the tissue.

Animals and other organisms

Policy information about studies involving animals; ARRIVE guidelines recommended for reporting animal research

Laboratory animals	Male and female Gsta4+/- mice were obtained from the Mutant Mouse Regional Resource Centers (MMRRC) (https://www.mmrrc.org/catalog/sds.php?mmrrc_id=11713). CBA/CaJ mice were purchased from the Jackson Laboratory (Bar Harbor, ME).
Wild animals	NA
Field-collected samples	NA
Ethics oversight	All animal experiments were conducted under protocols approved by the University of Florida Institutional Animal Care and Use Committee.

Note that full information on the approval of the study protocol must also be provided in the manuscript.
